# Gastroenteritis Outbreaks Caused by Norovirus GII.17, Guangdong Province, China, 2014–2015

**DOI:** 10.3201/eid2107.150226

**Published:** 2015-07

**Authors:** Jing Lu, Limei Sun, Lin Fang, Feng Yang, Yanling Mo, Jiaqian Lao, Huanying Zheng, Xiaohua Tan, Hualiang Lin, Shannon Rutherford, Lili Guo, Changwen Ke, Li Hui

**Affiliations:** Guangdong Provincial Center for Disease Control and Prevention, Guangzhou, China (J. Lu, L. Sun, L. Fang, F. Yang, Y. Mo, J. Lao, H. Zheng, X. Tan, H. Lin, L. Guo, C. Ke, L. Hui);; Guangdong Provincial Institution of Public Health, Guangzhou (J. Lu, H. Lin);; Centre for Environment and Population Health, Brisbane, Queensland, Australia (S. Rutherford)

**Keywords:** norovirus, GII.17, genotype, outbreaks, gastroenteritis, viruses, China

## Abstract

In the past decade, the most prevalent norovirus genotype causing viral gastroenteritis outbreaks worldwide, including China, has been GII.4. In winter 2014–15, norovirus outbreaks in Guangdong, China, increased. Sequence analysis indicated that 82% of the outbreaks were caused by a norovirus GII.17 variant.

Norovirus infection is a leading cause of nonbacterial gastroenteritis outbreaks in industrialized and developing countries (*1*,*2*). On the basis of amino acid identity in viral protein 1, noroviruses can be divided into at least 6 genogroups (GI–GVI). GI and GII infect humans and can be further classified into genotypes; at least 9 genotypes belong to GI and 22 belong to GII (*3*). During the past decade, most reported norovirus outbreaks were caused by GII.4 norovirus (*4*,*5*). New variants of GII.4 have emerged approximately every 2–3 years and have caused norovirus gastroenteritis pandemics globally (*6*). Since 1999, the major circulating genotype in mainland China has been GII.4, accounting for 64% of all genotypes detected (*7*). In winter 2014–15, norovirus outbreaks in Guangdong Province, China, increased. Sequence analyses showed that the major cause of continuous gastroenteritis outbreaks in the region was a rarely reported norovirus genotype: GII.17.

## The Study

In China, according to the National Public Health Emergency Contingency Plan, an outbreak with a cluster of at least 20 acute gastroenteritis cases (meeting the Kaplan criterion) within 3 days must be reported to Guangdong Provincial Center for Disease Control and Prevention. Samples from each outbreak are first tested for norovirus (Norovirus RT-PCR Kit; Shanghai ZJ Bio-Tech Co., Ltd., Shanghai, China) and for intestinal bacteria at the local Centers for Disease Control and Prevention. The norovirus-positive specimens are delivered to the Guangdong Provincial Center for Disease Control and Prevention for further genotyping.

From each outbreak, 5–10 samples are randomly selected for sequencing. Testing with One-Step RT-PCR (QIAGEN, Valencia, CA, USA) is performed with region C–specific primer, as previously described (*8*). The positive PCR products are sequenced, and norovirus genotypes are determined by using the Norovirus Automated Genotyping Tool (http://www.rivm.nl/mpf/norovirus/typingtool) or blastn (http://blast.ncbi.nlm.nih.gov/Blast.cgi). The major norovirus genotype causing each outbreak is defined as 1 genotype detected in >80% samples from the outbreak.

**Figure 1:**
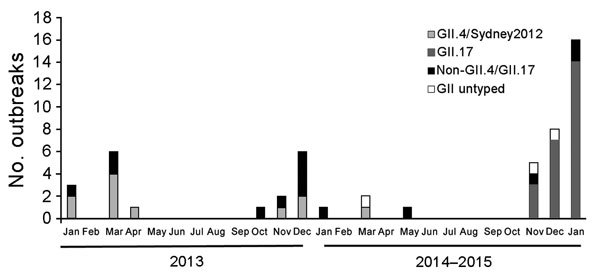
Norovirus outbreaks in Guangdong, Province, China, January 2013–January 2015.

From January 2013 through January 2015, a total of 52 norovirus outbreaks were reported and were associated with 4,618 clinical cases; of these, 14 outbreaks were associated with ≈100 clinical cases. Of the 52 outbreaks, 44 (85%) occurred in schools and colleges, 5 (9.6%) in factories, and 3 (5.7%) in kindergartens. In Guangdong Province, norovirus outbreaks are highly seasonal; most (96%) outbreaks are reported from November through March (Figure 1). In late 2014, an increase in the number of norovirus outbreaks was noted. From November 2014 through January 2015, a total of 29 identified outbreaks were associated with 2,340 cases compared with 9 outbreaks and 949 cases the previous winter (2013–14).

Samples from 46 (88%) of the 52 outbreaks were successfully genotyped. GII norovirus was detected in samples from 96% of the outbreaks. From January 2013 through October 2014, the most common genotype found was GII.4/Sydney/2012, which was detected in samples from 48% of the outbreaks. Genotype GII.17 was first detected in the city of Guangzhou in November 2014 and thereafter spread rapidly (Technical Appendix Figure 1). From November 2014 through January 2015, GII.17 norovirus outbreaks were reported in 10 cities of Guangdong Province and represented 83% (24 of 29) of all outbreaks. In contrast, during 2013 and 2014, norovirus outbreaks caused by GII.4/Sydney/2012 were reported in only 5 cities in Guangdong.

**Figure 2:**
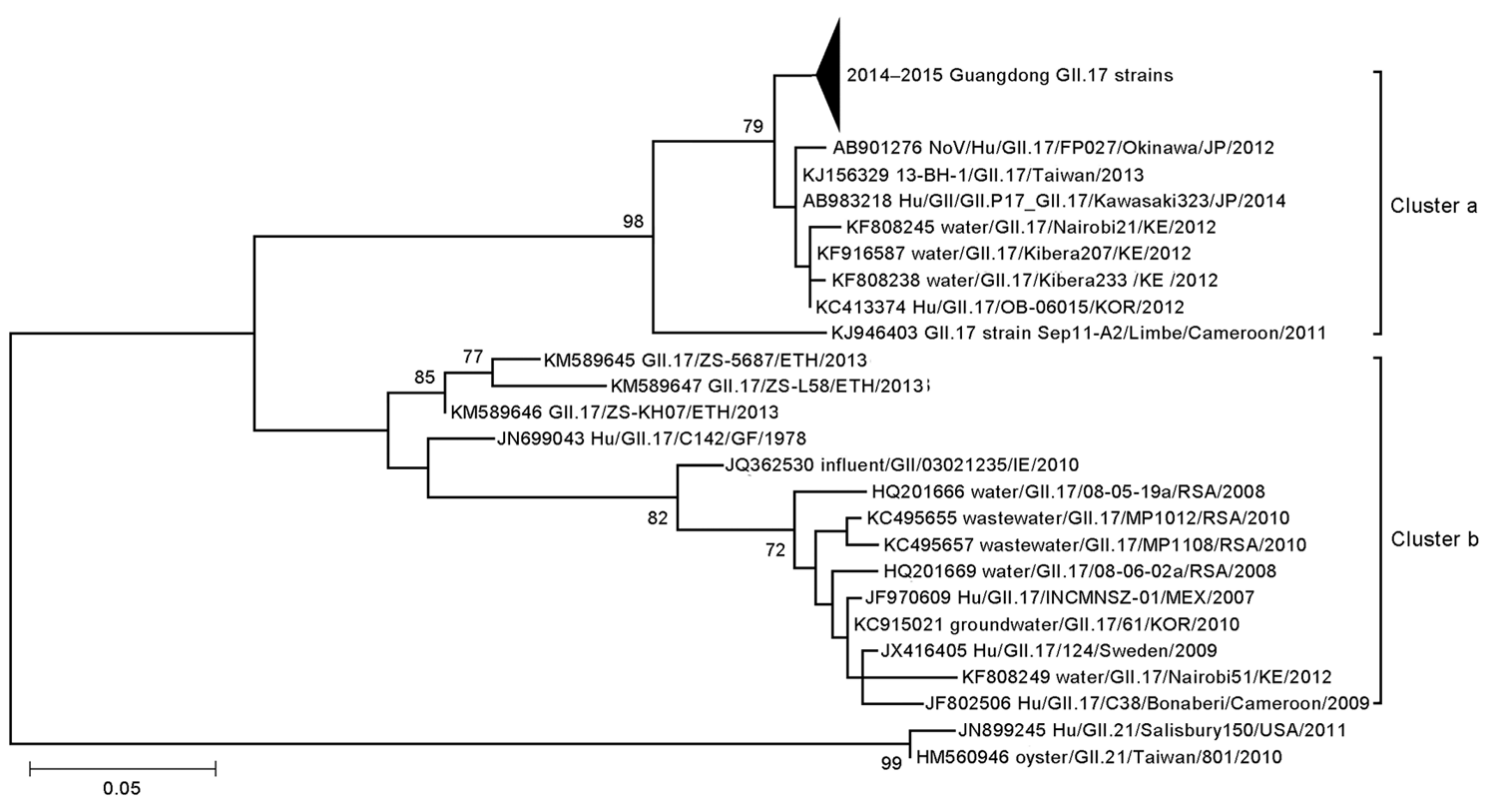
Phylogenetic tree of noroviruses based on the 282-bp region of the capsid N terminus/shell gene. Nucleotide sequences were analyzed by using the maximum-likelihood method. Supporting bootstrap values >70 are shown. The subtrees of GII.17 detected in Guangdong Province, China, during 2014–2015 were compressed. GII.21 genotype strains were used as outgroups. Scale bar indicates nucleotide substitutions per site. Sequences of 24 reference norovirus strains are included. Arrowhead represents number of strains from Guangdong, 2014–2015. ETH, Ethiopia; GF, French Guiana; IE, Ireland; JP, Japan; KE, Kenya; KOR, Korea; RSA, The Republic of South Africa; MEX, Mexico.

The nucleotide sequences of the norovirus GII.17 strains from Guangdong have been deposited in GenBank (accession nos. KP718638–KP718738). For phylogenetic analysis, representative strains from Guangdong were compared with GII.17 reference strains from the GenBank database. On the basis of region C sequences, genotype GII.17 could be divided into 2 major clusters (a and b). GII.17 strains collected from Guangdong during 2014–2015 norovirus outbreaks all clustered together and belonged to cluster b, the cluster to which all strains identified after 2011 belonged. Sequence comparison suggested that the strains most closely related to Guangdong GII.17 were from neighboring regions (e.g., Taiwan, Korea, and Japan) and from groundwater in Kenya (Figure 2).

## Conclusions

Outbreaks of nonbacterial gastroenteritis in Guangdong Province, China, during winter 2014–15 were caused by a rare norovirus, genotype GII.17. Previous epidemiologic data suggest that in the past 2 years, GII.4/Sydney/2012 has been the major circulating norovirus genotype worldwide (*4*,*5*,*9*). This GII.4 variant was first detected in Australia in March 2012 and was subsequently detected in France, New Zealand, Japan, the United Kingdom, the United States, and Hong Kong, and led to increased norovirus activity globally (*5*). In China, the GII.4/Sydney/2012 strain, first reported in October 2012, caused increased sporadic cases in the city of Shanghai (*10*). In early 2013 in Guangdong, GII.4/Sydney/2012 was the predominant norovirus genotype detected in norovirus outbreaks, while other genotypes including GII.3, GII.6, GI.2, GI.3, and GII.12 were occasionally detected. In winter 2013–14, detection of GII.4/Sydney/2012 decreased while detection of GII.3 and GII.6 increased. Norovirus genotype GII.17 was detected in the outbreak that occurred on November 18, 2014. Compared with GII.4/Sydney/2012, this variant of GII.17 displayed a high epidemic activity; in only 2 months, an increased number of related outbreaks were reported in 10 cities (Figure 1).

Sequence comparison with archived GII.17 strains from GenBank suggests that the GII.17 genotype identified in Guangdong is a newly emerged variant, differing from GII.17 strains detected before 2011. The recent detection of this new variant in samples from patients with sporadic cases in several regions of Asia (e.g., Korea, Japan, and Taiwan) and from groundwater in Kenya (*11*) suggests that this variant of GII.17 has circulated in a wide range of areas in recent years. For GII.17, most (66 [83%] of 80) sequences from the GenBank database are restricted to region C, the short conserved sequences of the N terminus of the capsid gene. This conserved region has been widely used for genotyping strains (*12*) and phylogenetic studies (*13*). To include more reference strains and to illustrate the relationship between GII.17 from Guangdong and other regions, we mainly used region C for phylogenetic analyses in this study. Similarly, phylogenetic analysis based on the nearly full length of capsid sequences also showed that the newly emerged GII.17 variant in Guangdong clustered with the strains from Japan and Taiwan in 2013 and 2014 and differed from GII.17 strains detected before 2011 (Technical Appendix Figure 2). 

In conclusion, a norovirus genotype GII.17 variant emerged in winter 2014–15 and caused outbreaks in multiple cities in Guangdong Province, China. The distribution of GII.17 genotype among patients with sporadic cases of gastroenteritis remains unknown. In future studies, epidemiologic and virologic surveillance should be broadened to better clarify virologic, clinical, and epidemiologic patterns of this newly emerged norovirus.

**Technical Appendix.** Distribution of GII.17 norovirus outbreaks and associated clinical cases in Guangdong Province, China, 2014–2015, and phylogenetic tree of noroviruses
