# Supplementary material for: Gastroenteritis Outbreaks Caused by Norovirus GII.17, Guangdong Province, China, 2014–2015
Source: Emerg Infect Dis. 2015 Jul;21(7):1240–2. doi: 10.3201/eid2107.150226 (PMC4480401; doi:10.3201/eid2107.150226)
Supplement: Supplementary file 1 — Technical Appendix. Distribution of GII.17 norovirus outbreaks and associated clinical cases in Guangdong Province, China, 2014–2015, and phylogenetic tree of noroviruses [file 15-0226-Techapp-s1.pdf]

DOI: http://dx.doi.org/10.3201/eid2107.150226

Gastroenteritis Outbreaks Caused by Norovirus GII.17, Guangdong Province, China, 2014–2015

Technical Appendix

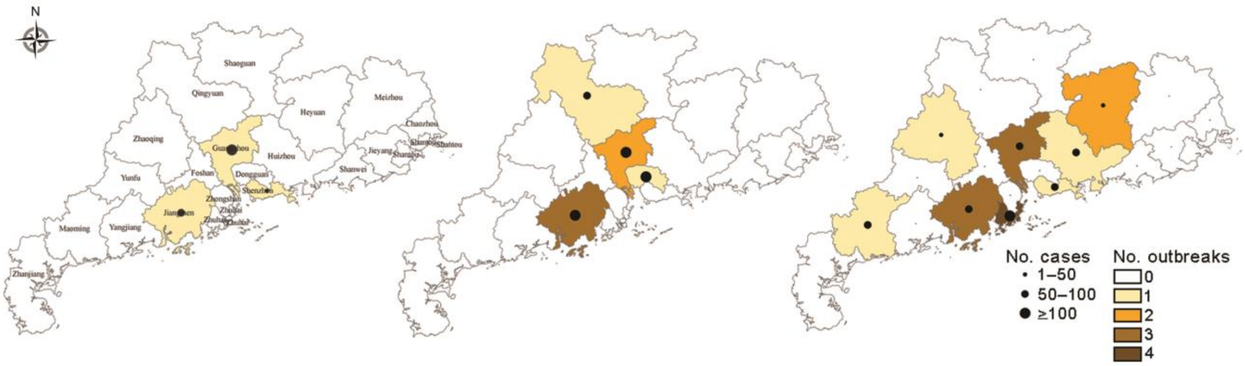

Technical Appendix Figure 1. Distribution of GII.17 norovirus outbreaks and associated clinical cases in Guangdong Province, China, showing November (left), December 2014 (middle), and January 2015 (right).

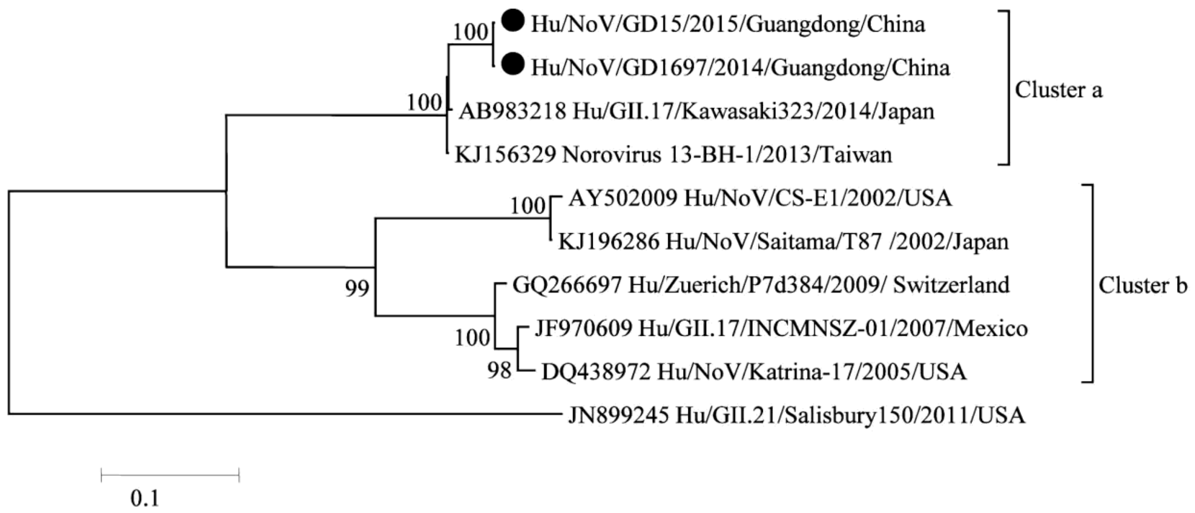

Technical Appendix Figure 2. Phylogenetic tree of noroviruses based on the nearly full length (1,486 bp) of the capsid. Nucleotide sequences were analyzed by using the maximum-likelihood method. Supporting bootstrap values of >70 are shown. GII.17 strains in Guangdong Province, China, 2014–2015, were marked with solid circles. GII.21 genotype strains were used as outgroups. Scale bar indicates nucleotide substitutions per site.
